# Topology optimization design of three-dimensional multi-material and multi-body structure based on irregular cellular hybrid cellular automata method

**DOI:** 10.1038/s41598-022-09249-y

**Published:** 2022-04-04

**Authors:** Xiaolei Deng, Hongxi Chen, Qiyue Xu, Fan Feng, Xingyi Chen, Xiaowen Lv, Xiaoliang Lin, Ting Fu

**Affiliations:** 1 Key Laboratory of Air-Driven Equipment Technology of Zhejiang Province, Quzhou University, Quzhou, 324000 China; 2 Key Laboratory of Metallurgical Equipment and Control Technology, Ministry of Education, Wuhan University of Science and Technology, Wuhan, 430081 China; 3 Hubei Key Laboratory of Mechanical Transmission and Manufacturing Engineering, Wuhan University of Science and Technology, Wuhan, 430081 China

**Keywords:** Engineering, Mathematics and computing

## Abstract

In recent years, Hybrid Cellular Automata Method (HCAM) has been successfully applied to solve structural topology optimization problems. However, there was no report on HCAM research of three-dimensional composite structure composed of multiple materials and multiple bodies. Therefore, in this paper, three-dimensional non-cube cells of irregular size (such as tetrahedral cells with adaptive changes inside length) and Finite Element Method (FEM) are introduced to extend HCAM, which is better and more flexibly to fit complex geometric shapes. Furthermore, a better structure configuration of multi-material and multi-body structure is obtained. The typical example study showed that the proposed topology optimization method could effectively remove the redundant materials of multi-material and multi-body structure, and the optimized structure configuration could still meet the requirements of the original condition after geometric reconstructed. Thus it provided a reference for the intelligent design of other products.

## Introduction

Topology optimization was a method to optimize the structure in design area according to the given external load and boundary conditions on the premise of satisfying the constraints such as stress, balance and displacement^1,2^. At present, continuum topology optimization methods mainly include variable density method, progressive structure optimization method, homogenization method and cellular automata method, etc.. These methods have been paid attention by many scholars. Zhang et al.^3^ used variable density method to conduct Finite Element Analysis and topology optimization design for a new energy bus seat bracket. They compared and verified the scaffolds before and after optimization, and found that the weight of the optimized scaffold was not only lighter, but also the mechanical properties were obviously improved. Zuo et al.^4^ proposed an Ordered Multi-material SIMP Interpolation Method to solve the problem of multi-material topology optimization. In this method, Power functions with scaling and translation coefficients were introduced to perform interpolation calculation on the elastic modulus and cost characteristics of multi-material. Therefore, the topology optimization configuration can be obtained without additional variables. Hoang et al.^5^ proposed a concurrent topology optimization approach based on adaptive geometric components and applied it to the three-dimensional (3D) printing technology suitable for the design and manufacture of honeycomb composite materials. Lambe et al.^6^ proposed a topology optimization method combining continuous material field with adaptive mesh refinement, which used different design and analysis grids to avoid islands of materials in the topology configuration, and adopted Helmholtz-type density filter to avoid some unnecessary small features in the design and analysis of mesh refinement. Banh et al.^7,8^ used topology optimization method to study multiphase material and multiple materials plate structure problem, and then they presented an effective non-homogeneous multi-material topology optimization paradigm for functionally graded structures considering both cracked and non‐cracked cases^9^.

In recent years, HCAM has attracted a large number of scholars. It is simple to understand, has good convergence, high computational efficiency and reduces numerical instability. It can be used to solve multi-objective problems and provides a new analytical idea and technical means for the effective topology optimization design of the structure^10,11^. Tovar et al.^12^ have applied HCAM in the optimization design of vehicle collision mitigation, energy collector shell and blast mitigating armor structures based on a novel method for designing crashworthy structures with controlled energy absorption and harmonic vibration loading method^13^. Aiming at the problem of topology optimization of nonlinear structures with large deformation, Guo et al.^14^ proposed a HCAM based multi-domain continuum structure topology optimization method, and then introduced the strain-based and multi-domain topology optimization algorithm into HCAM to study vehicle collision avoidance under large deformation^15^. In order to solve the static load requirements under normal operating conditions and energy absorption requirements targeting passive safety in crash events existing in the process of vehicle design, Aulig et al.^16^ applied the proportional energy weighting method based on priority approach to HCAM, and applied it to the optimization design of the conceptual structure of industrial vehicle body. Afrousheh et al.^17^ improved the search efficiency of HCAM by introducing the variable neighborhood radius concept, so as to achieve the purpose of improving the energy absorption of vehicle structure under high collision. This method realized an intelligent search strategy of improved HCAM. Da et al.^18^ used HCAM to carry out topology optimization research on materials with extreme properties, and obtained the optimal topology configuration of the design object without obtaining sensitivity information through energy homogenization. Deng et al.^19^ applied the HCAM in the thermal topology optimization design of the spindle structure under temperature-structural field coupling condition, after topology optimization, the spindle configuration not only reduced the material, but also improved its thermal characteristics. However, this method is only used for solving multi-field coupling topology optimization of 2D plane structures, and has not been carried out for the topology optimization of 3D structures. Jia et al.^20^ proposed a topology optimization method for two-scale non-uniform microstructures based on HCAM, and applied the method to the optimization design of 2D and three-dimensional two-scale non-uniform microstructures.

In general, the research on 3D multi-material and multi-structure topology optimization using HCAM has not been reported. This paper proposes a 3D topology optimization method based on irregular cells of HCAM to study the 3D continuum structure with a variety of materials, which was aim to obtain the optimal structure configuration. Finally, the effectiveness of the method is verified by comparing with other algorithms. This paper discusses the new idea and gives the numerical calculation process based on the method to provide a reference for product intelligent design.

## 3D irregular cell HCAM model

### 3D HCAM topology optimization model based on irregular cell

**Figure 1:**
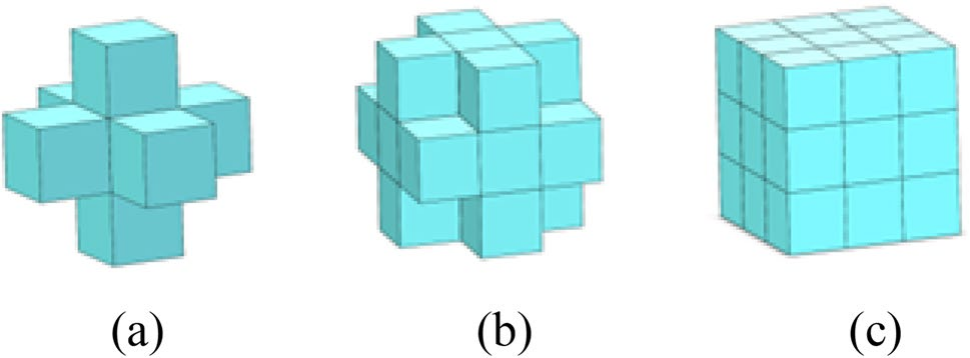
Neighborhoods of regular cell in a 3D lattice^11^. (**a**) 3D von Neumann, $\hat{N}{ = 6}$ (**b**) 3D Radial, $\hat{N}{ = 18}$ (**c**) 3D Moore, $\hat{N}{ = 26}$.

Now 3D HCAM is commonly based on regular cube cells, which is shown in Fig. 1. Figure 1a is a 3D von Neumann type neighbor cell ($\hat{N}{ = 6}$). Only six immediate neighbors are considered, and these neighboring cells have common surfaces with the central cell. Figure 1b shows a 3D Radial type neighbor cell ($\hat{N}{ = 18}$). The neighbor cell with common edge or surface with the central cell is considered to be the neighbor cell of the central cell. Figure 1c is a 3D Moore type neighbor cell ($\hat{N}{ = 26}$), and any cell with a common surface or edge or vertex with the central cell can be considered as the neighbor cell of the central cell.

**Figure 2:**
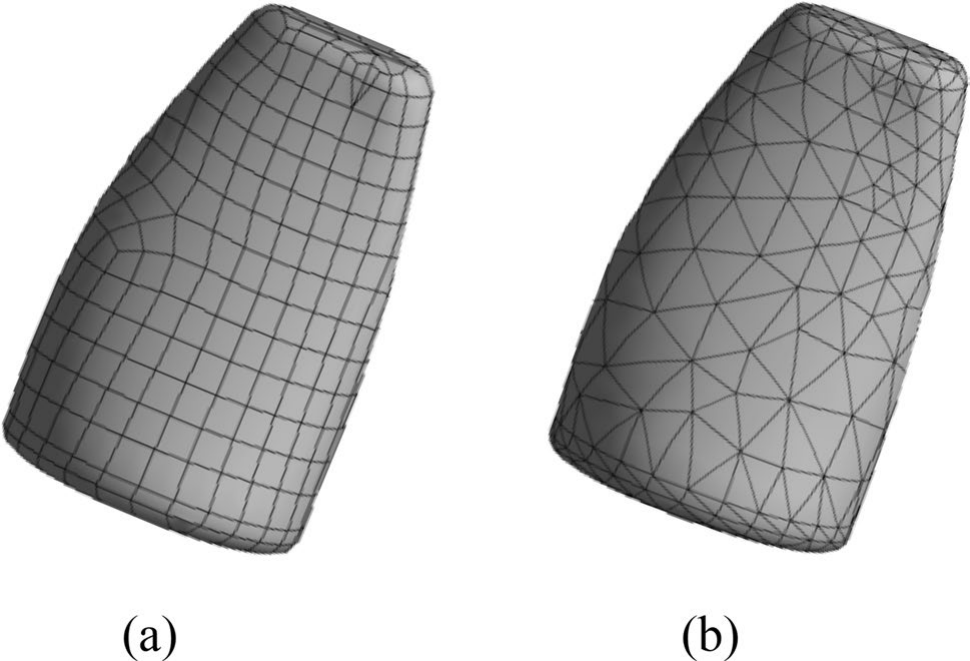
Shape meshed by different cell types. (**a**) shape meshed by cube cells (**b**) shape meshed by tetrahedral cells.

**Figure 3:**
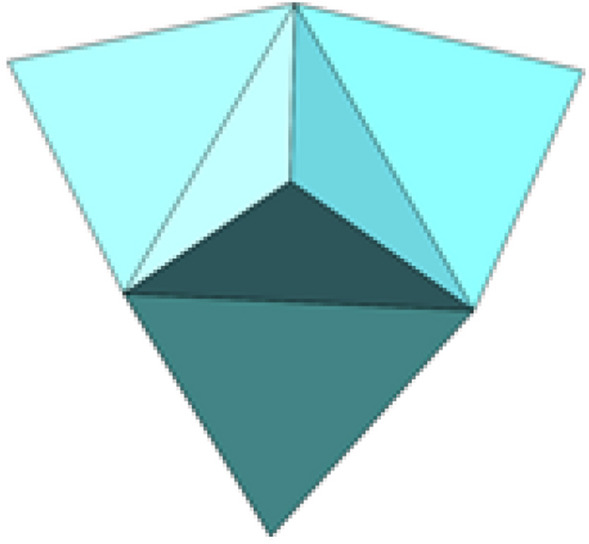
Neighborhoods of irregular cell in 3D tetrahedral von Neumann.

In many cases, cube cells cannot cover the design area uniformly. In practical engineering analysis and design, irregular cells can be used to deal with complex geometric figures. In key areas of concern, cells need to be locally refinement. In this paper, the traditional cube cells of regular size are expanded to three-dimensional non-cube cells of irregular size (such as tetrahedral cells with adaptive side length), which could fit complex geometric shapes better and more flexibly. Thus, better solutions were obtained when solving topological configurations, as shown in Fig. 2. In some places, the shape of non-cube cells appears, even though the cube cells is set, as shown in Fig. 2a. At the same time, introducing the idea of local grid refinement in complex model fitting, a sharp change in the stress concentration or strain area for local cell refinement, which do not need to use the whole structure of fine cell. And it can be used to avoid excessive number of cellular automata and cause cell with the difficulty of the mapping between Finite Element mesh, speed up the calculation, increase the authenticity of the results. As shown in Fig. 3, only four adjacent neighboring cells for the tetrahedral von Neumann type neighboring cells (the number of neighboring cells is 4) are considered. And these adjacent cells had common surfaces with the central cell. The number of neighboring cells for tetrahedral von Neumann type is 2 less than that of cubic von Neumann type, which can effectively reduce the calculation amount of topology optimization.

According to the theory of HCAM in literature^11^, for discrete position *i* and discrete time *t*, the cellular state set ${{a_{i}}} (t)$ of the HCAM model can be expressed as follows:

1 $${{a_{i}}} (t) = \left\{ {{a_{i}^{1}} (t),{a_{i}^{2}} (t), \ldots ,{{a_{i}^{J}}} (t)} \right\}$$

where, *J* is the number of cellular states; ${a_{i}^{J}} \left( t \right)$ is the *J*th state defined for discrete time *t* and discrete position *i*.

The state at a time (*t*+1) under each cell is jointly determined by its state at time *t* and the state of its neighboring cell. The *J*th cell’s status update rule can be expressed as

2 $${a_{i}^{J}} (t + 1) = {R_{i}^{J}} \left\{ {{a_{i}} (t),{{a_{i + \Delta 1}}} (t), \ldots a_{{i + \hat{N}}} (t)} \right\}$$

where, ${a_{i + \Delta 1}} (t)$,…,${a_{{i + \hat{N}}}} (t)$ is the neighborhood cell of cellular *i*; $\hat{N}$ is the number of cells in the neighborhood of a cell; ${R_{i}^{J}}$ is the local update rules of cell *i*, which is the same for all positions.

The neighbor cell query rules of 3D tetrahedral irregular cell HCAM are as follows: all the cells and their nodes are taken out at first, and then the nodes of each cell are compared. If two cells have three identical nodes, the two cells are defined as neighbors.

In the iterative process of topology optimization using HCAM, the design variable $x_{i} (t)$ of each cell can be defined as cell density, geometric size, elastic modulus, etc., and the state field variable $S_{i} (t)$ of each cell can be defined as stress, strain, strain energy density or a function of them, Then, the state ${{{\varvec{a}}_{i}}} (t)$ of the cell at time *t* can be expressed as

3 $${{\varvec{a}}_{i}} (t) = \left[ \begin{aligned} x_{i} (t) \hfill \\ S_{i} (t) \hfill \\ \end{aligned} \right]$$

The design variable $x_{i} (t)$ is determined according to the material model. In this paper, the relative density of the element is taken as the design variable, and the definition of the field variable $S_{i} (t)$ depends on the optimization problem to be solved. Then, in a discrete region, the strain energy *E* of the whole structure is

4 $$E = \sum\limits_{i = 1}^{N} {U_{i} v_{i} }$$

where, $U_{i}$ is the strain energy density stored due to deformation in cellular *i* in CA; $v_{i}$ is the volume of cellular *i*.

5 $$\left\{ {\begin{array}{*{20}l} {{U_{i}} = \frac{1}{2}{{\varvec{\sigma}}^{T}} {\varvec{\varepsilon}}} \hfill \\ {{\varvec{\varepsilon}} = [{\varepsilon_{x}} ,{\varepsilon_{y}}, {\varepsilon_{z}} ,{\gamma_{xy}} , {\gamma_{yz}} ,{\gamma_{xz}} ]^{T} } \hfill \\ {{\varvec{\sigma}} = [{\sigma_{x1}} ,{\sigma_{y1}} ,{\sigma_{z1}} ,{\tau_{xy}} ,{\tau_{yz}} ,{\tau_{xz}} ]^{T} } \hfill \\ \end{array} } \right.$$

where, $\varepsilon_{x}$, $\varepsilon_{y}$, $\varepsilon_{z}$, $\gamma_{xy}$, $\gamma_{yz}$, $\gamma_{xz}$ are the *x*-direction strain, *y*-direction strain, *z*-direction strain and shearing strain of the node respectively; $\sigma_{x1}$, $\sigma_{y1}$, $\sigma_{z1}$ are the normal stresses in *x*-direction, *y-*direction and *z*-direction respectively; $\tau_{xy}$, $\tau_{yz}$, $\tau_{xz}$ are the shear stress on surfaces *xy*, *yz* and *xz*.

The strain energy density is the local rigidity index of the structure. Thus, the strain energy density is used as the field variable in this paper, and the optimization criterion refers to the uniform distribution of the strain energy density. From this standard, the CA state can be defined as

6 $${{\varvec{a}}_{i}} (t) = \left[ \begin{gathered} x_{i} (t) \hfill \\ U_{i} (t) \hfill \\ \end{gathered} \right]$$

The topology optimization design of the structure is carried out by using HCAM, and the definition of strain energy density $U_{i} (t)$ can be determined by the specific optimization objective function. The objective of optimization is to minimize the difference between the mean value of cell strain energy density $\overline{U}_{i}$ and the set value of state field variable, the optimization problem can be expressed by Eq. (7)

7 $$\left\{ {\begin{array}{*{20}l} {\min \left| {e_{i} } \right| = \left| {\overline{U}_{i} - U^{*}} \right|} \hfill \\ {{\text{s.t.}}\;0 < x_{{{\text{min}}}} \le x_{i} \le 1,\quad i = 1,2, \ldots ,N} \hfill \\ \end{array} } \right.$$

where, $e_{i}$ is the error signal; $U^{*}$ is the set value of strain energy density. In the equation, there is a requirement of lower limit $x_{{{\text{min}}}}$, so that the singular matrix can be avoided in the process of FEA.

At this point, the state of the cell, the strain energy of the whole structure and the strain energy density $U_{i} (t)$ are the same as the regular cell model, but the $\overline{U}_{i} (t)$ of the strain energy density of cell *i* is changed, which is expressed by Eq. (8):

8 $$\overline{U}_{i} = \frac{{\frac{{S_{i} }}{S} \times U_{i} + \sum\limits_{j = 1}^{{\hat{N}}} {\frac{{S_{j} }}{S}U_{j} } }}{{\hat{N} + 1}}$$

where, *U*_*j*_ is the strain energy density of adjacent cell *j* which is adjacent to cell *i*; *S* is the average area of each cell in CA; *S*_*i*_ is the area of cell *i*; *S*_*j*_ is the area of neighboring cell *j* which is adjacent to cell *i*.

In order to expand the application range of HCAM optimization model and accelerate the convergence of topology optimization design results, so as to adapt to structural topology optimization with fixed and variable loads, the target value of strain energy density U^*^ in the process of topology optimization is calculated by using Eq. (9)

9 $$U^{*} = aif \times \frac{{E_{t} }}{{V_{0} }}$$

where, *aif* is weight coefficient; *V*_0_ is the volume of the original structure; *E*_*t*_ is the total strain energy of the structure when iteration to *t* times.

### Convergence criterion

In the iterative process of topology optimization, whether the structure meets the criteria of convergence is determined by the type of design rules used in the constantly updated design variables. The change of structure mass, $\Delta M(t)$ is taken as the convergence criterion, then the structure mass is expressed as

10 $$\left\{ {\begin{array}{*{20}l} {M(t) = \sum\limits_{i = 1}^{N} {\rho_{i} (t)} v_{i} = \sum\limits_{i = 1}^{N} {x_{i} (t)\rho } v_{i} } \hfill \\ {\Delta M(t) = M(t) - M(t - 1)} \hfill \\ {0 \le x_{i} (t) \le 1} \hfill \\ \end{array} } \right.$$

where, $M(t)$ is the total mass of the structure from iteration to *t* times; $\rho_{i} (t)$ is the density of cell *i* when iteration to *t* time; $\rho$ is solid material density. When the cell relative density $x_{i} (t)$ is 1, the cell is composed of solid material; When the cell relative density $x_{i} (t)$ is 0, the cell can be considered to have no material.

When $\Delta M(t)$ in mass goes to zero, that means there’s no change in mass. Then it is determined that the optimization process has reached the convergence standard. At the same time, in order to avoid the partial phenomenon that small changes in quality may occur in the application process of HCAM and cause large changes in structure, and to reduce the accidental uncertainty, the convergence criterion uses the average change of two consecutive iterations to determine

11 $$\frac{{\left| {\Delta M(t)} \right| + \left| {\Delta M(t - 1)} \right|}}{{2M_{0} }} \le \varepsilon$$

where, *M*_0_ is the total mass of the initial structure. Mass ratio *F* is proposed to reflect the specific optimization degree of the current iteration number structure, then the mass ratio *F*(*t*) at time *t* can be expressed as follows

12 $$F\left( t \right) = \frac{M\left( t \right)}{{M_{0} }} = \frac{{\sum\limits_{i}^{N} {\rho_{i} \left( t \right)v_{i} } }}{{M_{0} }}{ = }\frac{{\sum\limits_{i}^{N} {\rho_{i} \left( t \right)v_{i} } }}{{\sum\limits_{i}^{N} {\rho v_{i} } }}$$

### Local control rule

The material distribution in the design domain is determined by the local control rule, which seeks to minimize the difference $e_{i} \left( t \right)$ between the state field variable $\overline{U}_{i} (t)$ and the set value of the state field variable $U^{*}$ when iteration to *t* times, so that the calculation result tends to the target value. Linear control rule is adopted in this paper to express the change value of the relative cell density of the material as follows

13 $$\Delta x_{i} \left( t \right) = f(e_{i} \left( t \right)) = C_{p} \times e_{i} \left( t \right)$$

where, $f(e_{i} \left( t \right))$ is a local control rule, $C_{p}$ is a linear weight coefficient, usually a normal number.

## Topology optimization design of 3D monomer structure

In order to verify the effectiveness and universality of HCAM based on tetrahedral irregular cell topology optimization algorithm, topology optimization design is carried out by using different three-dimensional monomer structures and working cases.

### Topology optimization design of L-shaped bracket

**Figure 4:**
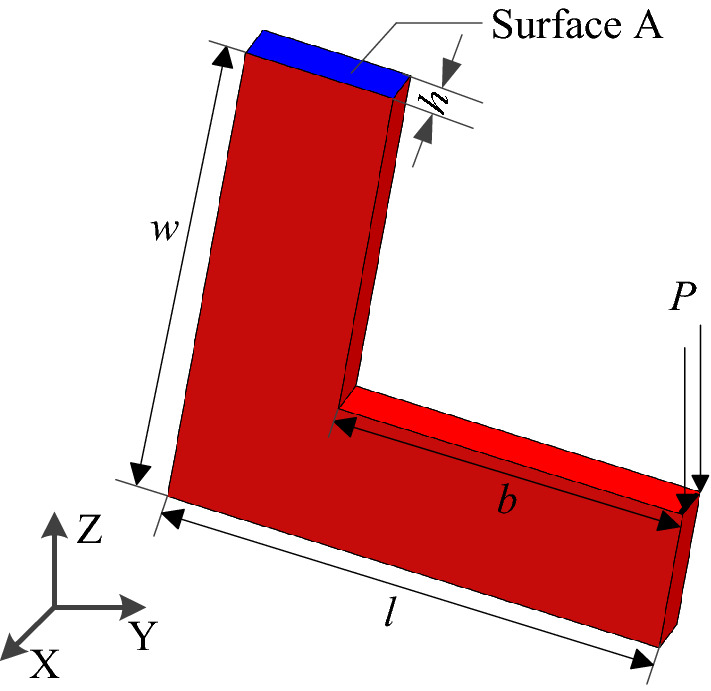
L-shaped bracket structure model.

The L-shaped bracket structure is shown in Fig. 4. The bottom length *l* = 10 mm, height *w* = 10 mm, thickness *h* = 1 mm, cross arm length *b* = 7 mm. Material elastic modulus *E*_0_ = 200GPa, Poisson’s ratio *μ* = 0.3, density $\rho = 7850{\text{ kg/m}}^{3}$, yield limit $\sigma_{s} = 250{\text{ MPa}}$. The constraints and loads are shown in Fig. 4. A fixed constraint is imposed on the Surface A of the L-shaped bracket, and the concentrated load $P = 6.5{\text{ N}}$ along the negative direction of the Z-axis is set on the right edge of the transverse arm. In this case study, tetrahedral elements are used as the cell shape of HCAM, and tetrahedral von Neumann type is selected as the neighboring cells, when calculating by FEM, selecting the mesh element SOLID186, a total of 66,005 tetrahedral cells (grids) are generated by dividing the structure, and the cell size is consistent with the grid size. The optimization goal is to remove 30% of the material, The initial design variable $x_{i} (0) = 1$, the convergence error $\varepsilon = 10^{ - 4}$, the weight coefficient $aif = 1.75$, the local control rules are linear control rules, the linear weight coefficient $C_{p} = 1$.

**Figure 5:**
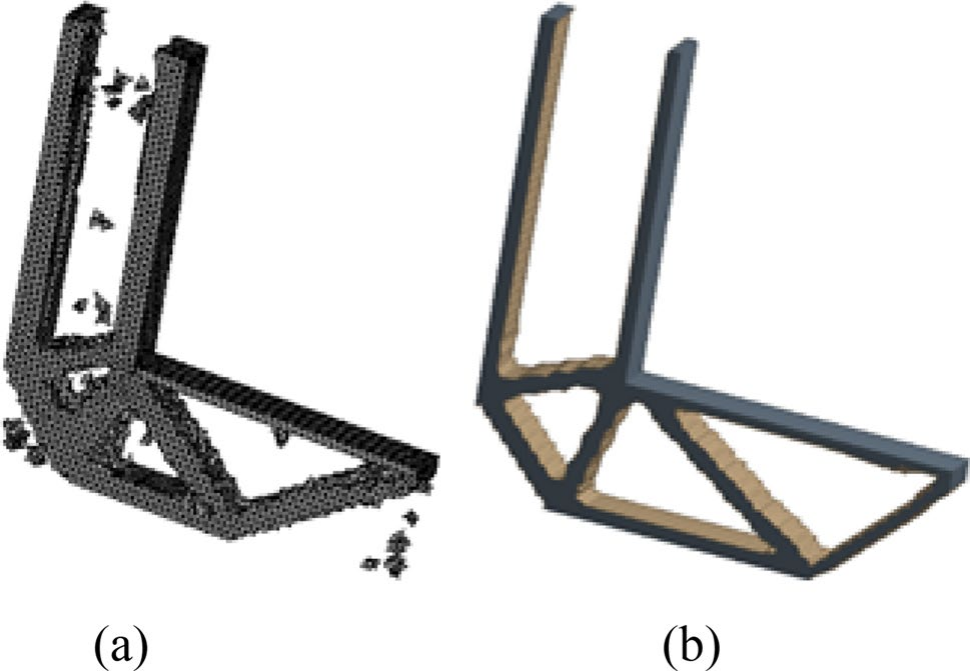
Comparison of optimization results with different optimization algorithms. (**a**) HCAM optimization result (**b**) FEM optimization result.

Then, the number of iterations to solve the result is 38 based on HCAM calculation results, and the final topology optimization configuration obtained is shown in Fig. 5a. It is very similar to the topology optimization configuration obtained by ANSYS Topological Optimization Module in Fig. 5b.

**Figure 6:**
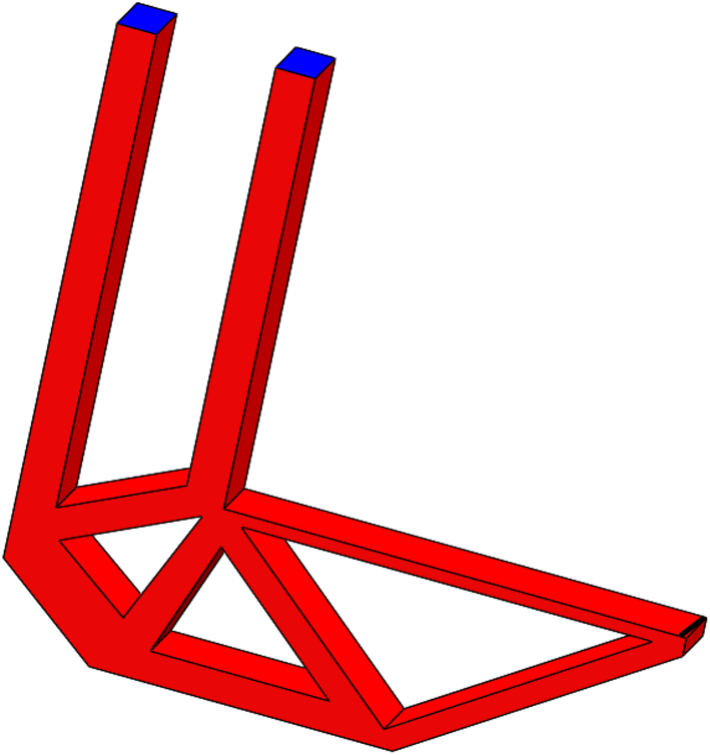
CAD software reconstructed model.

The topology optimization configuration obtained above is output to CAD software for geometric reconstructed of the model. The reconstructed model basically kept the original topology structure of the optimization result, and the results are shown in Fig. 6.

**Figure 7:**
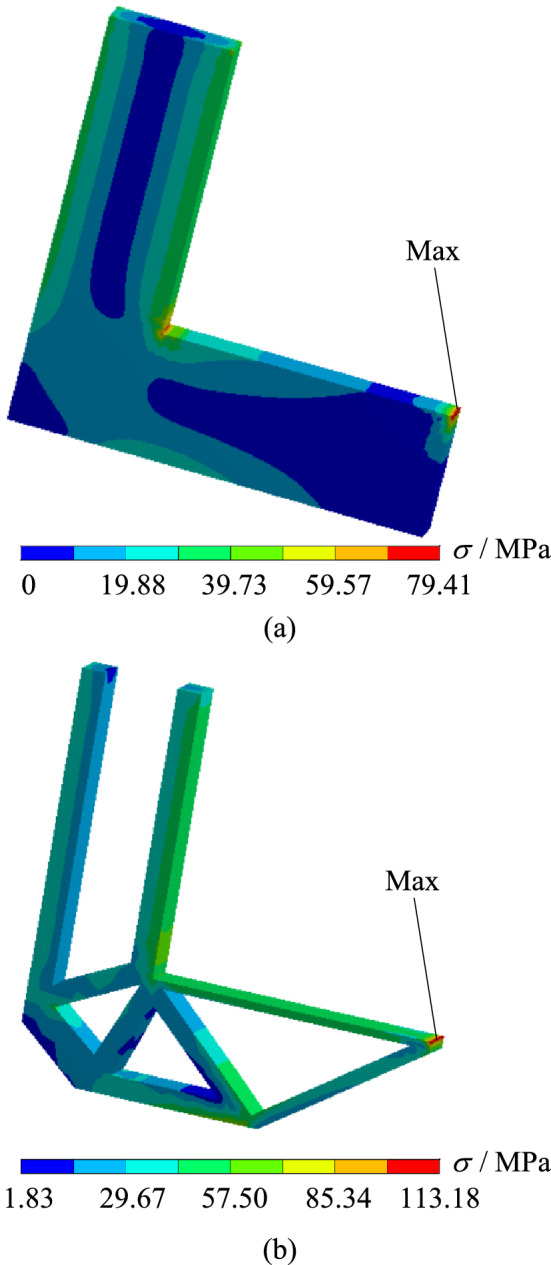
Comparison of stress contour figures before and after optimization. (**a**) Stress contour figure of the original model (**b**) Stress contour figure of the reconstructed model.

**Figure 8:**
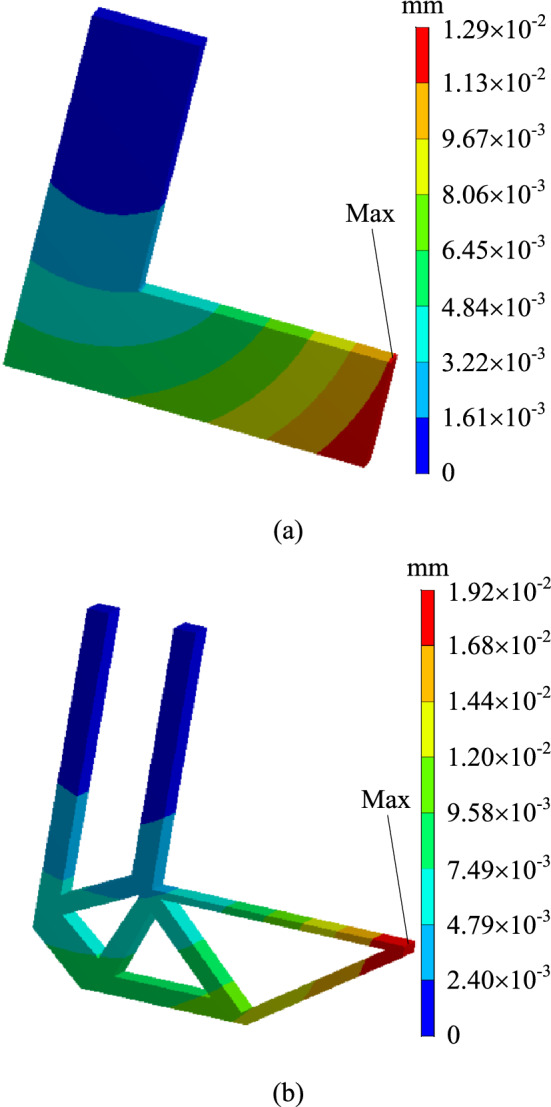
Comparison of displacement contour figures before and after optimization. (**a**) Displacement contour figure of the original model (**b**) Displacement contour figure of the reconstructed model.

In order to verify whether the geometrically reconstructed model could meet the requirements of working conditions, static simulation calculation is carried out under the same boundary conditions. At the same time, the stress and displacement comparison results are obtained as shown in Figs. 7 and 8. As can be seen from the stress contour figures in Fig. 7, the maximum stress position of the original model and the reconstructed model is basically the same, both appearing on the right side of the transverse arm, The maximum stress of the original model is about 79.41 MPa, and that of the reconstructed model is about 113.18 MPa. When 70% of the material is removed, the maximum stress increases by about 42.6%. However, the safety factor *n* is 2, which could still meet the strength requirements. Meanwhile, as can be seen from the displacement contour figures in Fig. 8, the maximum displacement of the original model appeared below the right-most end of the L-shaped bracket cross arm, and the maximum displacement of the reconstructed model appears at the upper right end of the L-shaped bracket cross arm, which are $1.29 \times 10^{ - 2} {\text{ mm}}$ and $1.92 \times 10^{ - 2} {\text{ mm}}$ respectively.

For the examples of L-shaped support structure under the same working conditions, the mass ratio *F* is set to 30%, 50% and 70% respectively, and the results are compared with those obtained by FEM. The results are shown in Table 1.

**Table 1:**
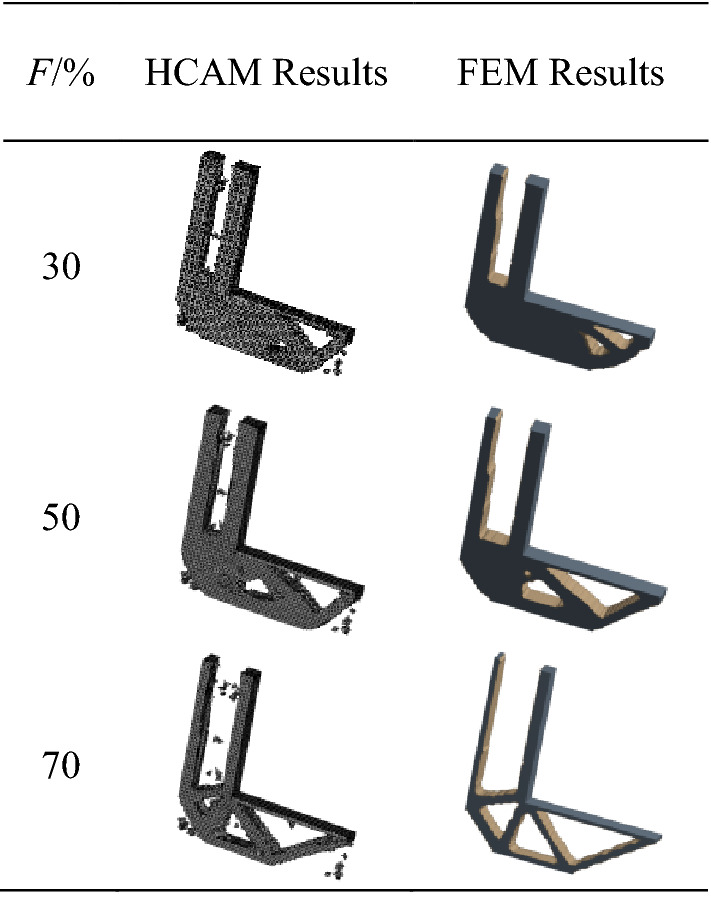
Comparison of optimization results of different mass ratios.

**Figure 9:**
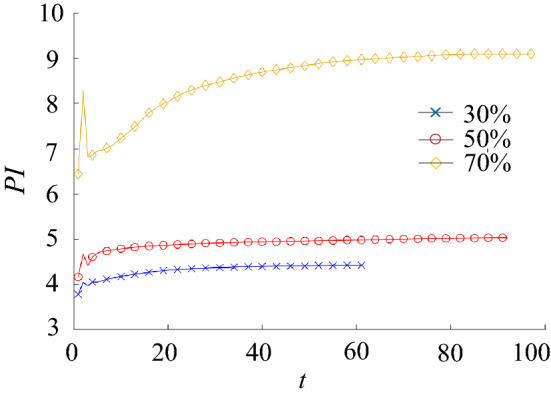
The performance index history curve under different material removal ratio condition.

The performance indexes of each generation are calculated, and the fitting curve of HCAM performance indicators is obtained, as shown in Fig. 9. It could be seen that the topology optimization results of the three mass ratios tend to converge. As reported in the literature^11^, hundreds of thousands of iterations for same conventional topology optimization methods are required to achieve convergence. When using the HCAM presented in this work, the number of iterations can be reduced to about 60 iterations.

By comparing the optimization results of HCAM and FEM under different mass ratios, it could be seen that the topology optimization configuration of 3D structures with different mass ratios could be obtained effectively using HCAM. And it is very similar to that obtained by FEM.

### Topology optimization design of the regular triangular yoke plate structure

**Figure 10:**
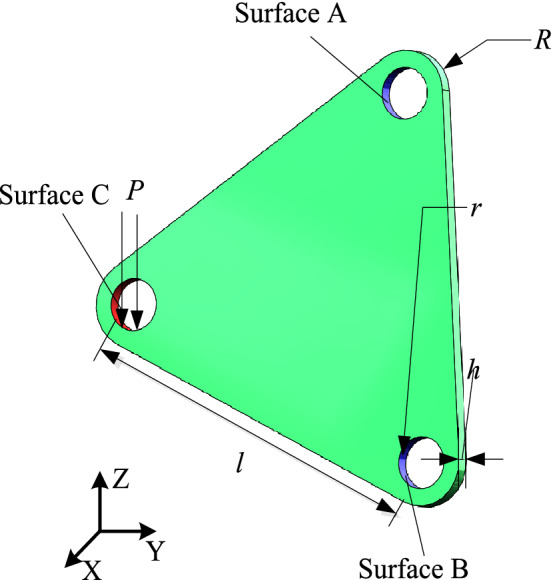
Regular triangular yoke plate.

The regular triangular yoke plate structure is shown in Fig. 10, where its side length *l *= 60 mm, the thickness *h *= 2 mm, fillet radius *R *= 10 mm, round hole radius *r *= 5 mm. The modulus of elasticity of the material *E*_0_ = 200 GPa, Poisson’s ratio *μ *= 0.3, density $\rho = 7850{\text{ kg/m}}^{3}$, and yield limit $\sigma_{s} = 250{\text{ MPa}}$. The constraints and load settings are shown in Fig. 10. Fixed constraints are imposed on the Surface A and Surface B of the circular hole Surface. And concentrated load $P = 800{\text{ N}}$ in the negative direction along the Z axis is set on the Surface C of the circular hole Surface. Meanwhile, tetrahedral cell are used as the cell shape of HCAM, and tetrahedral von Neumann type is selected as the neighboring cells. During the Finite Element calculation, the mesh element is chosen as SOLID186 (high-order 3D 20-node solid structure element), and a total of 108,297 tetrahedral grids (cells) are generated after dividing the structure, and the cell size is consistent with the grid size so that map to each other. The optimization goal is to remove 50% of the material. The initial design variable $x_{i} (0) = 1$, the convergence error $\varepsilon { = 10}^{{{ - }4}}$, the weight coefficient $aif = 0.5$, the local control rule adopts the linear control rule, and the linear weight coefficient $C_{p} = 0.5$.

.

**Figure 11:**
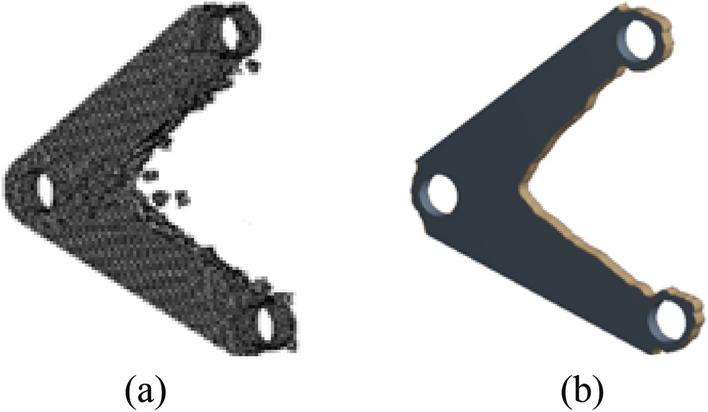
Comparison of optimization results with different optimization algorithms. (**a**) HCAM optimization result (**b**) FEM optimization result.

After HCAM calculation, the number of iterations is 26, and the final topology optimization configuration obtained is shown in Fig. 11a, which is very similar to the topology optimization configuration obtained by using the ANSYS topology optimization module in Fig. 11b.

**Figure 12:**
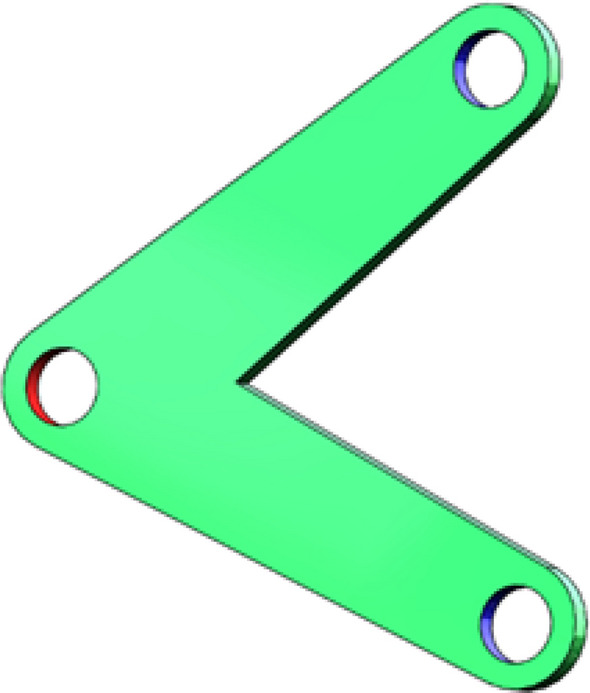
CAD software reconstructed model.

The topology optimization configuration obtained above is output to CAD software for geometric reconstructed of the model. The reconstructed model basically kept the original topology structure of the optimization result, and the results are shown in Fig. 12.

**Figure 13:**
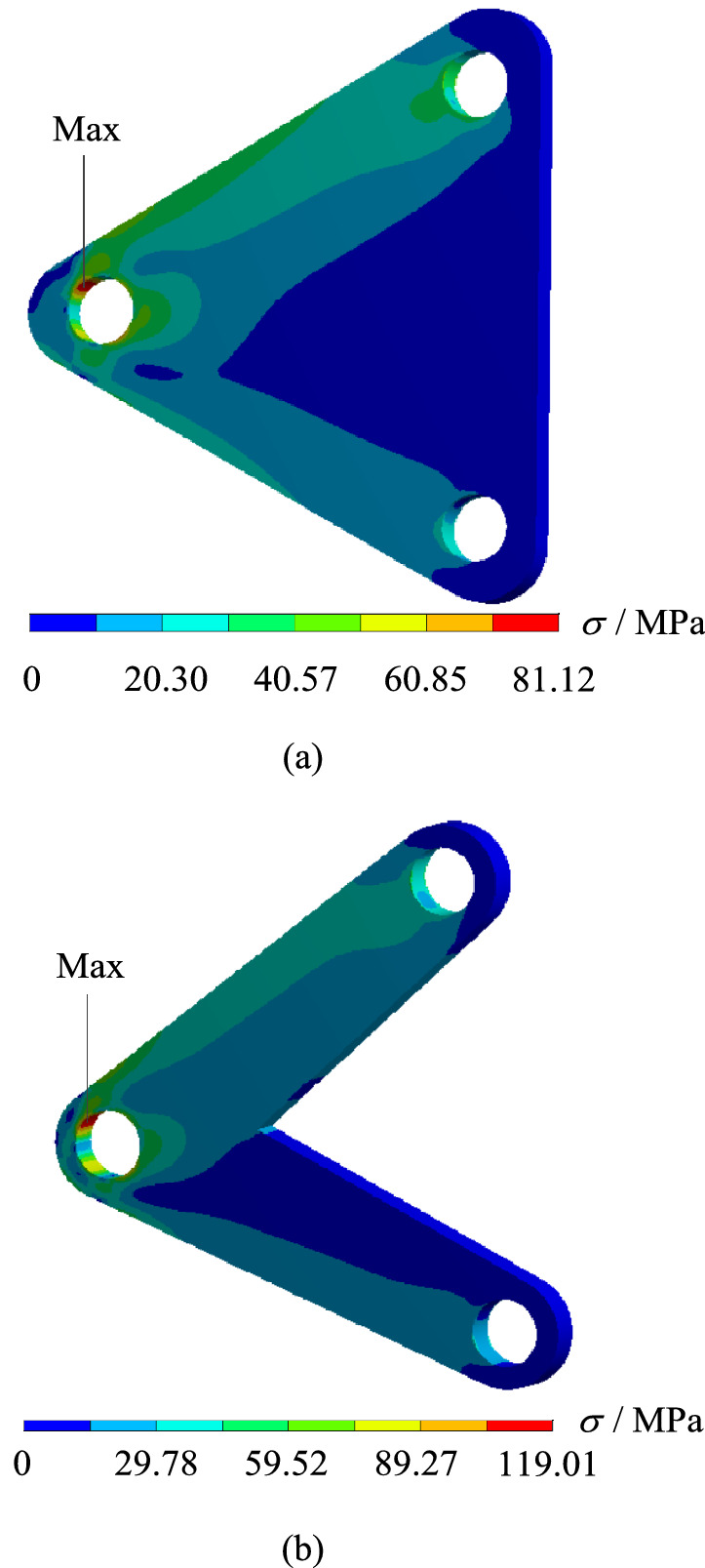
Comparison of stress contour figures before and after optimization.(**a**) Stress contour figure of the original model (**b**) Stress contour figure of the reconstructed model.

**Figure 14:**
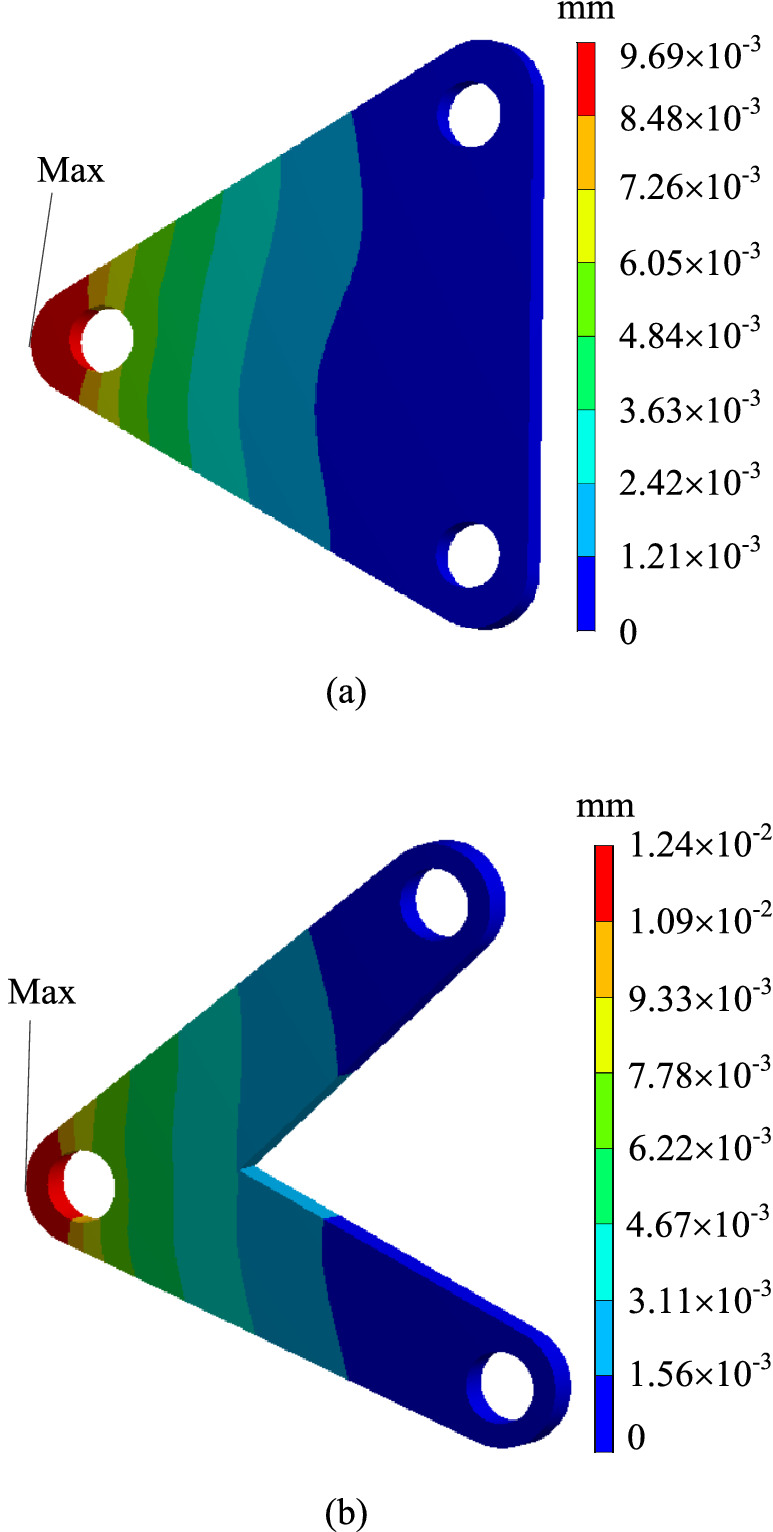
Comparison of displacement contour figures before and after optimization.(**a**) Displacement contour figure of the original model (**b**) Displacement contour figure of the reconstructed model.

In order to verify whether the geometrically reconstructed model could meet the requirements of working conditions, static simulation calculation is carried out under the same boundary conditions, and the stress and displacement comparison results are obtained as shown in Figs. 13 and 14. As can be seen from the stress contour figures in Fig. 13, the maximum stress position of the original model and the reconstructed model is basically the same, both appearing on the upper left of the Surface C of the circular hole, the maximum stress value of the original model is about 81.12 MPa, and that of the reconstructed model is about 119.01 MPa, when 50% of the material is removed, the maximum stress increased by about 46.7%, but the yield limit of the material is 250 MPa, and the safety factor *n* is 2, which could still meet the strength requirements. Meanwhile, as can be seen from the displacement contour figures in Fig. 14, the maximum displacement position of the original model and the reconstructed model is basically the same, both appearing on the rounded corner surface outside the circular hole Surface C. The maximum displacement of the initial scheme is $9.69 \times 10^{ - 3} {\text{ mm}}$, and the maximum displacement of the reconstructed structure is $12.40 \times 10^{ - 3}$ mm.

The material removal ratio *F* of the triangular yoke plate structure is set to 30%, 70% and 80% of the original structure respectively, and HCAM and FEM are used to carry out the comparative analysis of its topology optimization. The results are shown in Table 2.

**Table 2:**
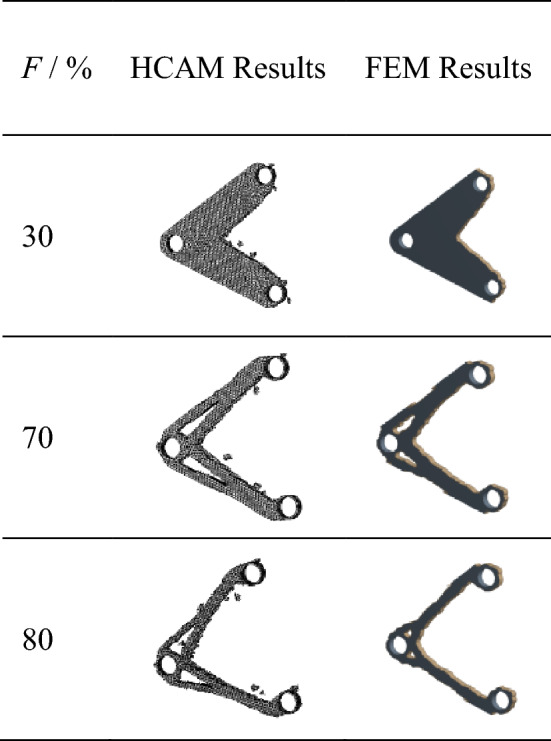
Comparison of optimization results of different mass ratios of triangular coupling plates.

**Figure 15:**
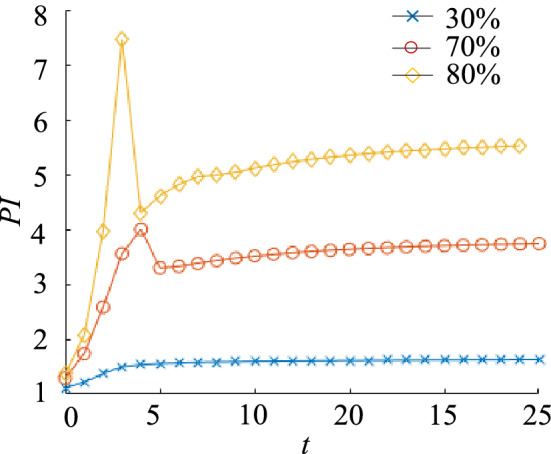
The performance index history curves under different material removal ratio condition.

The performance indexes of each generation are calculated, and the fitting curve of HCAM performance indicators is obtained, as shown in Fig. 15. It could be seen that the topology optimization results of the three mass ratios tend to converge less than 25 iterations. By comparing the optimization results, it could be seen that the topology optimization configuration are very similar obtained by HCAM and FEM.

## 3D multi-material and multi-body HCAM model

### Finite element analysis model for contact problem

**Figure 16:**
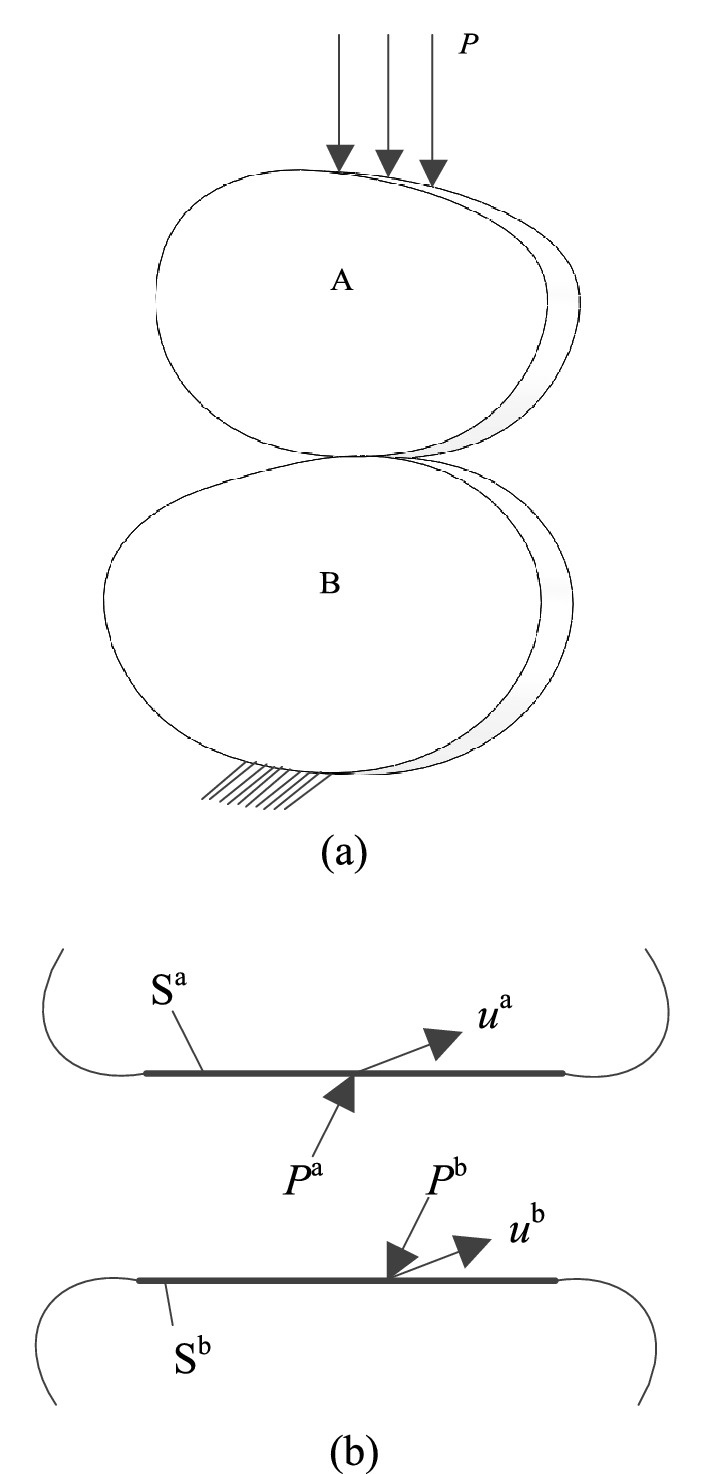
Schematic diagram of contact problem between two objects. (**a**) Force diagram of two contact bodies (**b**) Force and displacement diagram of the contact surfaces.

In 3D multi-material and multi-body analysis, the contact relationship between objects is unavoidable, and FEM is the most effective method to deal with the contact problem. As shown in Fig. 16a, object A and object B are in contact. Assuming that S^a^ is the contact surface and S^b^ is the target surface (as shown in Fig. 16b), the boundary on object B is fixed, external load *P* is applied on object A, and the two points on which surface S^a^ and S^b^ contact each other are called contact point pairs. According to literature^21^, the basic Finite Element equations of object A and object B are

14 $$\left\{ {\begin{array}{*{20}c} {[K^{{\text{a}}} ]\{ u^{{\text{a}}} \} = \{ R^{{\text{a}}} \} + \{ P^{{\text{a}}} \} } \\ {[K^{{\text{b}}} ]\{ u^{{\text{b}}} \} = \{ R^{{\text{b}}} \} + \{ P^{{\text{b}}} \} } \\ \end{array} } \right.$$

In the formula, $[K^{{\text{a}}} ]$ and $[K^{{\text{b}}} ]$ represent the rigid body matrices of object A and object B, $\{ u^{{\text{a}}} \}$ and $\{ u^{{\text{b}}} \}$ represent the nodal displacement vectors of object A and object B, $\{ R^{{\text{a}}} \}$ and $\{ R^{{\text{b}}} \}$ represent the contact force vectors of object A and object B, $\{ P^{{\text{a}}} \}$ and $\{ P^{{\text{b}}} \}$ represent the vectors of external forces acting on object A and object B.

In Eq. (14), $\{ u^{{\text{a}}} \}$, $\{ u^{{\text{b}}} \}$, $\{ R^{{\text{a}}} \}$ and $\{ R^{{\text{b}}} \}$ are unknown quantities. When both $[K^{{\text{a}}} ]$ and $[K^{{\text{b}}} ]$ are non-singular matrices, the flexibility equation of the contact point can be obtained

15 $$\left\{ {\begin{array}{*{20}c} {\{ u_{i}^{{\text{a}}} \} = \sum\limits_{j = 1}^{m} {[C_{ij}^{{\text{a}}} ]} \{ R_{j}^{{\text{a}}} \} + \sum\limits_{k = 1}^{{n^{{_{{\text{a}}} }} }} {[C_{ik}^{{\text{a}}} ]} \{ P_{k}^{{\text{a}}} \} } \\ {\{ u_{i}^{{\text{b}}} \} = \sum\limits_{j = 1}^{m} {[C_{ij}^{{\text{b}}} ]} \{ R_{j}^{{\text{b}}} \} + \sum\limits_{k = 1}^{{n^{{_{{\text{b}}} }} }} {[C_{ik}^{{\text{b}}} ]} \{ P_{k}^{{\text{b}}} \} } \\ \end{array} } \right.$$

where, *i*, *j* and *k* represent the node number, *i* = 1, 2…,*m*; *j* = 1, 2,…,*m*. *m* represents the number of node pairs, *n*^a^ and *n*^b^ represent the number of external force acting points of object A and object B respectively, $[C_{ij}^{{\text{a}}} ]$ and $[C_{ij}^{{\text{b}}} ]$ represents the ${3} \times {3}$ order flexibility sub-matrices at point *i* on object A and object B in different directions of *x*, *y* and *z*, respectively, which are derived from the unit force at point *j*. And it also defines as follow

16 $$\{ u_{i}^{{\text{a}}} \} = [u_{ix}^{{\text{a}}} \, u_{iy}^{{\text{a}}} \, u_{iz}^{{\text{a}}} ]^{{\text{T}}}$$

17 $$\{ u_{i}^{{\text{b}}} \} = [u_{ix}^{{\text{b}}} \, u_{iy}^{{\text{b}}} \, u_{iz}^{{\text{b}}} ]^{{\text{T}}}$$

The defined quantity $\{ u_{i}^{{\text{a}}} \}$, $\{ u_{i}^{{\text{b}}} \}$ represents the displacement vector of the contact point *i* on object A and object B, respectively. In the same way

18 $$\{ R_{j}^{{\text{a}}} \} = [R_{jx}^{{\text{a}}} \, R_{jy}^{{\text{a}}} \, R_{jz}^{{\text{a}}} ]^{{\text{T}}}$$

19 $$\{ R_{j}^{{\text{b}}} \} = [R_{jx}^{{\text{b}}} \, R_{jy}^{{\text{b}}} \, R_{jz}^{{\text{b}}} ]^{{\text{T}}}$$

The definition quantity $\{ R_{j}^{{\text{a}}} \}$, $\{ R_{j}^{{\text{b}}} \}$ represents the contact force vectors of the contact point *j* on object A and object B respectively. Define again

20 $$\{ P_{k}^{{\text{a}}} \} = [P_{kx}^{{\text{a}}} \, P_{ky}^{{\text{a}}} \, P_{kz}^{{\text{a}}} ]^{{\text{T}}}$$

21 $$\{ P_{k}^{{\text{b}}} \} = [P_{kx}^{{\text{b}}} \, P_{ky}^{{\text{b}}} \, P_{kz}^{{\text{b}}} ]^{{\text{T}}}$$

The definition quantity $\{ P_{k}^{{\text{a}}} \}$,$\{ P_{k}^{{\text{b}}} \}$ represents the external force vectors acting on node *k* on object A and object B respectively.

$\sum\limits_{k = 1}^{{n^{{_{{\text{a}}} }} }} {[C_{ik}^{{\text{a}}} ]} \{ P_{k}^{{\text{a}}} \}$ and $\sum\limits_{k = 1}^{{n^{{_{{\text{b}}} }} }} {[C_{ik}^{{\text{b}}} ]} \{ P_{k}^{{\text{b}}} \}$ represent the displacement vectors caused by external forces at the contact point *i* on object A and object B, respectively.

In the analysis of FEM in this paper, bonded type is adopted for the contact model of multi-material and multi-body structure, that is, relative sliding or separation between contact surfaces is not allowed. The contact element uses the 4-node CONTACT174 element, and the corresponding target element uses the 8-node TARGET170 element. A single “contact pair” consists of a target element and a contact element. The program uses a shared real constant to identify a “contact pair”. When establishing a “contact pair”, the target element and the contact element must specify the same real constant^22^.

### Convergence criterion of multi-material and multi-body structure

Whether a multi-material and multi-body structure achieves convergence criteria is also determined by the type of design rules used by the constantly updated design variables. Assuming that the multi-material and multi-body structure contains *N* objects, the convergence criterion can be taken as the change $\Delta M_{{{\text{Tot}}}} (t)$ of the total mass $M_{{{\text{Tot}}}} (t)$, which can also be determined according to the average change of two consecutive iterations

22 $$\left\{ {\begin{array}{*{20}l} {M_{{{\text{Tot}}}} (t) = \sum\limits_{Q = 1}^{N} {M_{Q} } } \hfill \\ {\Delta M_{{{\text{Tot}}}} (t) = M_{{{\text{Tot}}}} (t) - M_{{{\text{Tot}}}} (t - 1)} \hfill \\ {\frac{{\left| {\Delta M_{{{\text{Tot}}}} (t)} \right| + \left| {\Delta M_{{{\text{Tot}}}} (t - 1)} \right|}}{{2M_{{{\text{Tot}}}} (0)}} \le \varepsilon } \hfill \\ \end{array} } \right.$$

where, *Q* = 1, 2,…, *N*.

## Topology optimization of three-dimensional multi-material and multi-body structures

### Topology optimization design of cylinder-torus plate structure

**Figure 17:**
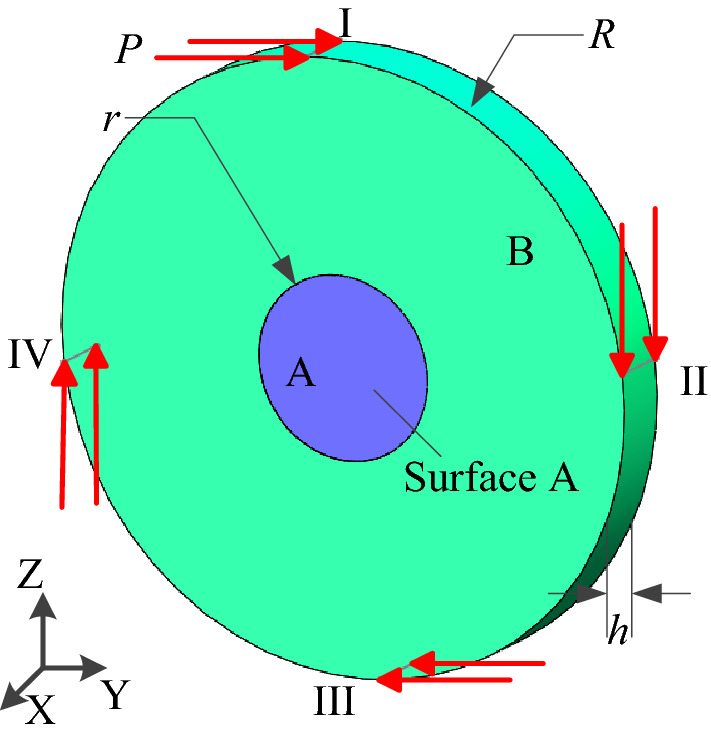
Cylinder-torus plate structure model.

The cylinder-torus plate assembly structure as shown in Fig. 17 is a multi-material and multi-body structure, which was consisted of a cylinder plate A with a radius *r* = 3 mm and an torus plate B with an outer diameter *R* = 10 mm. The outer perimeter of the cylinder plate A is in contact with the inner perimeter of the torus plate B. The thickness of the two plates *h* = 2 mm, the modulus of material elasticity of cylinder plate A $E_{0}^{{\text{a}}} = 71{\text{ GPa}}$, yield limit $\sigma_{s}^{{\text{a}}} = 280{\text{ MPa}}$; Poisson’s ratio $\mu^{{\text{a}}} = 0.33$, and the material density $\rho = 2770{\text{ kg/m}}^{3}$; Elasticity modulus of torus plate B $E_{0}^{{\text{b}}} = 200{\text{ GPa}}$, Poisson’s ratio $\mu^{{\text{b}}} = 0.3$, density $\rho = 7850{\text{ kg/m}}^{3}$, yield limit $\sigma_{s}^{{\text{b}}} = 250{\text{ MPa}}$. A fixed constraint is applied on the Surface A for the cylinder plate A. And a concentrated load $P = 30\;{\text{N}}$ is applied on the outer circumferential Surface I–IV of torus plate B. In this case study, tetrahedral elements are used as the cell shape of HCAM, and tetrahedral von Neumann type is selected as the neighboring cells. The cylindrical plate A is regarded as the rigid body, and the torus plate B as the flexible body. When calculating with FEM, select the mesh element as SOLID186 and contact type  as Bonded. The 3D 8-node Target170 element is used at the target surface, and the 3D 4-node Contect174 element is used at the contact surface, and constrain the contact surface between them to produce static friction only. There are 107,330 tetrahedral cells (grids) after dividing the cylinder-torus plate structure. The torus plate B is divided into 96,923 tetrahedral cells (grids), and the cell size is consistent with the Finite Element size. The torus plate B is set as the design domain, and the optimization objective is set to remove 60% of the material. The initial design variable $x_{i} (0) = 1$, the convergence error $\varepsilon = 10^{ - 3}$, the weight coefficient *aif* = 65,000, the local control rule is the linear control rule, the linear weight coefficient $C_{p} = 0.45$.

**Figure 18:**
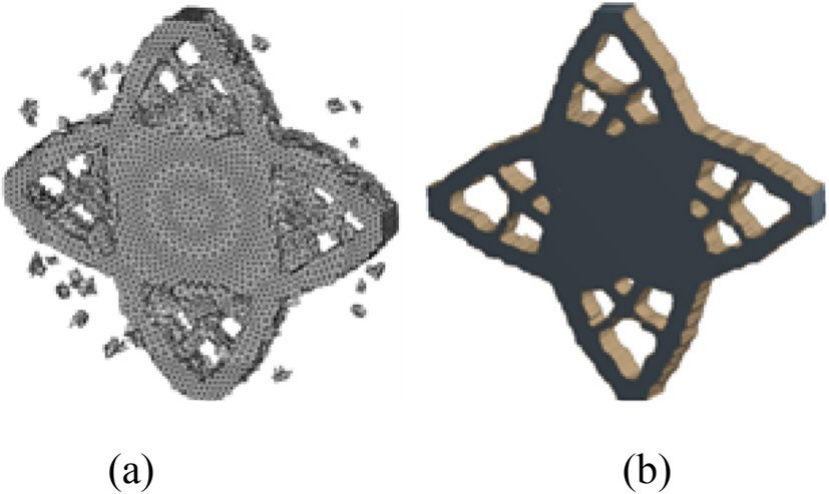
Comparison of optimization results with different optimization algorithms. (**a**) HCAM optimization result (**b**) FEM optimization result.

After HCAM calculation, the final iteration number of solving the result is 20 times. The final topology optimization configuration obtained is shown in Fig. 18a, which is very similar to the result obtained by ANSYS in Fig. 18b. The effectiveness and reliability of the proposed HCAM based tetrahedral irregular cell topology optimization algorithm for multi-material and multi-body are verified.

**Figure 19:**
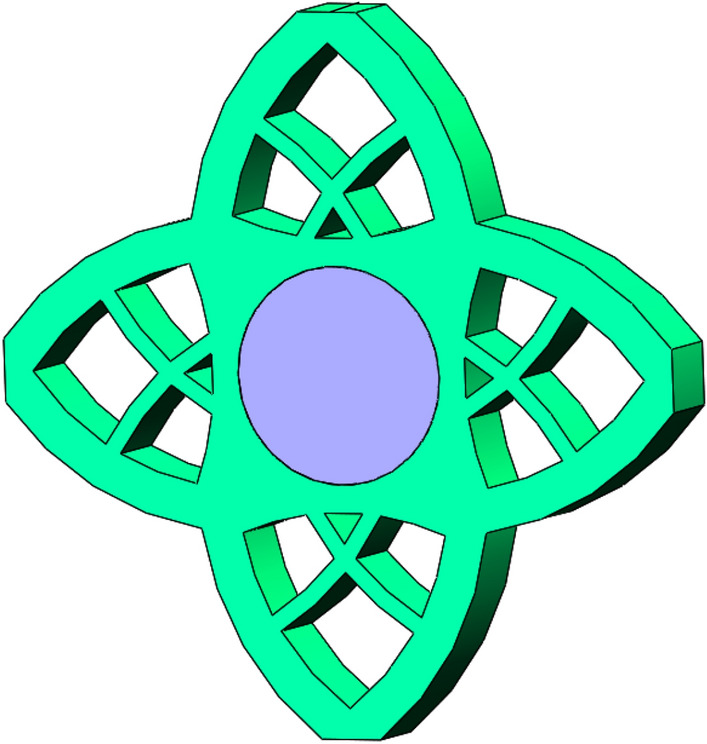
CAD software reconstructed model.

In this paper, the obtained multi-material and multi-body topology optimization configuration is output to CAD software for geometric reconstructed of the model. The reconstructed model basically kept the original topology structure of the optimization result, as shown in Fig. 19.

**Figure 20:**
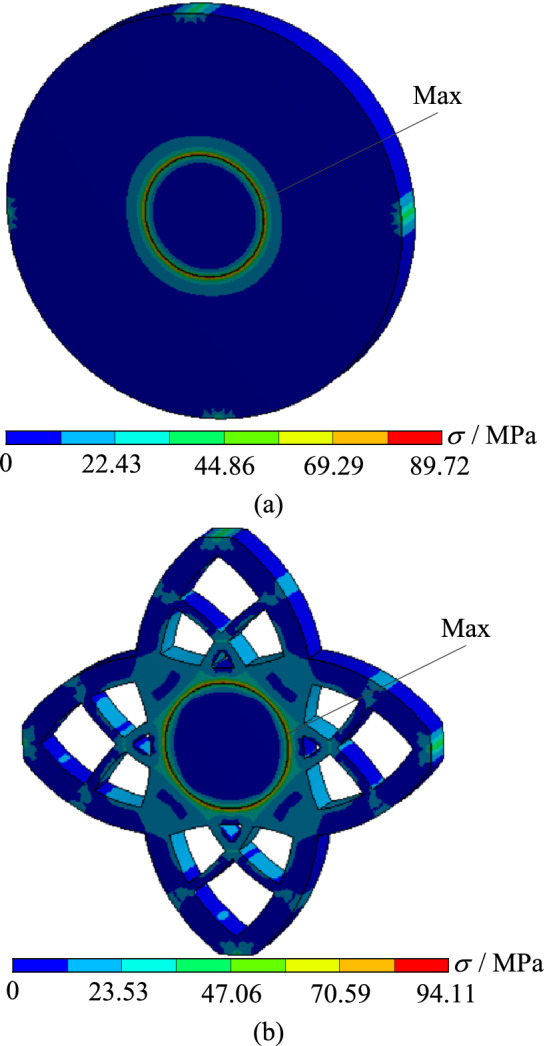
Comparison of stress contour figures before and after optimization. (**a**) Stress contour figure of the original model (**b**) Stress contour figure of the reconstructed model.

**Figure 21:**
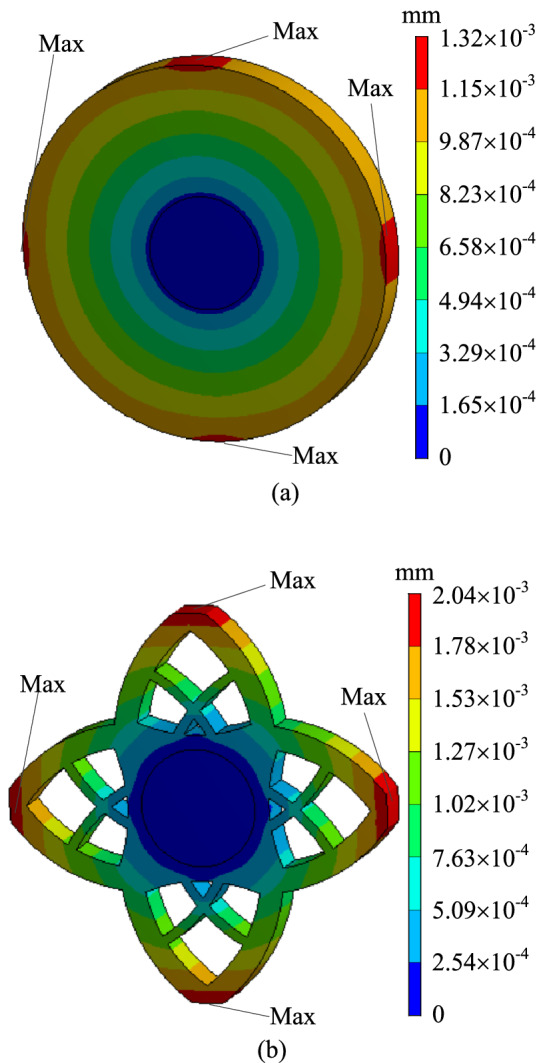
Comparison of displacement contour figures before and after optimization. (**a**) Displacement contour figure of the original model (**b**) Displacement contour figure of the reconstructed model.

In order to verify whether the geometrically reconstructed model can meet the requirements of working conditions, static simulation calculation is carried out under the same boundary conditions. Thus the stress and displacement comparison results are obtained, as shown in Figs. 20 and 21. As can be seen from the stress contour figures in Fig. 20, the maximum stress of both the original model and the reconstructed model appears at the contact position of the two plates. The maximum stress of the original model is about 89.72 MPa and that of the reconstructed model is about 94.11 MPa. When 60% materials are removed, the maximum stress increases by 4.9%. But the material yield limit of the torus plate B is 250 MPa, and the safety factor *n* is 2, which can still meet the strength requirements. At the same time, the maximum stress of cylinder plate A is 47.49 MPa, which is far lower than the material yield limit of cylinder plate A of 280 MPa. Figure 21 is the displacement contour figure of the structure before and after optimization, it can be seen from the figure that the maximum displacement positions of the original model and the reconstructed model are basically the same, both appearing near the place (I–IV) where the loads are applied, which are $1.32 \times 10^{ - 3} {\text{ mm}}$ and $2.04 \times 10^{ - 3} {\text{ mm}}$ respectively.

### Topology optimization design of support-connecting rod-support structure

**Figure 22:**
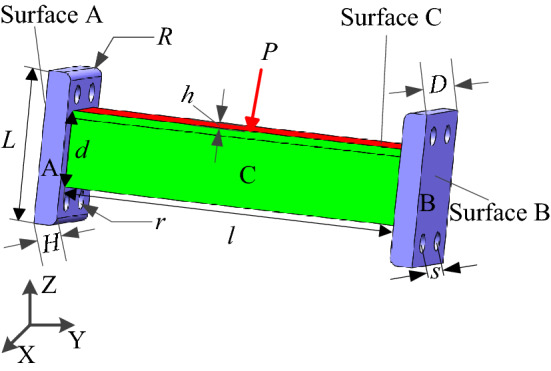
Support-connecting rod-support structure.

As shown in Fig. 22, the support-connecting rod-support multi-material and multi-body structure consists of two identical supports A and B and a connecting rod C of length *l* = 45 mm, width *d* = 10 mm and thickness *h* = 2 mm. On the support A and B, the thickness *H* = 2 mm, length *L* = 20 mm, width D = 15 mm, radius of fillet on the support R = 1 mm, round hole radius *r* = 2 mm, hole center distance *s* = 7 mm. The left and right end surfaces of connecting rod C are connected with the corresponding surfaces of supports A and B. The material elastic modulus of support A and B is $E_{0}^{{\text{a}}} = E_{0}^{{\text{b}}} = 71{\text{ GPa}}$, poison’s ratio $\mu^{{\text{a}}} = \mu^{{\text{b}}} = 0.33$, material density $\rho^{{\text{a}}} = \rho^{{\text{b}}} = 2770{\text{ kg/m}}^{3}$, yield limit $\sigma_{s}^{{\text{a}}} = \sigma_{s}^{{\text{b}}} = 280{\text{ MPa}}$. For supports C, the elasticity modulus of the material $E_{0}^{{\text{c}}} = 200{\text{ GPa}}$, poison’s ratio $\mu^{{\text{c}}} = 0.3$, density $\rho^{{\text{c}}} = 7850{\text{ kg/m}}^{3}$ and yield limit $\sigma_{s}^{{\text{c}}} = 250{\text{ MPa}}$. A load $P = 850{\text{ N}}$ is applied on the upper Surface C of the connecting rod C. And fixed constraint are applied on the Surface A and Surface B of the two supports, and only static friction is ensured between the support A and B with the connecting rod C.

In this case study, tetrahedral elements are used as the cell shape of HCAM, and tetrahedral von Neumann type is selected as the neighboring cells. The support A and B are taken as rigid body, connecting rod C is taken as a flexible body. The mesh element SOLID186 and contact type bonded are selected to calculate for FEM. The 8-node TARGET170 element is used at the target surface, and the 4-node Contect174 element is used at the contact surface. And constrain the contact surfaces between them to produce only static friction. A total of 116,111 tetrahedral cells are obtained by dividing the support-connecting rod-support boy structure into irregular cells. Among them, connecting rod C is divided into 80,342 tetrahedral cells (grids), and the cell size is consistent with the Finite Element size. Connecting rod C is set as the design domain and the optimization objective is set to remove 40% of the material. The initial design variable $x_{i} (0) = 1$, the convergence error $\varepsilon = 10^{ - 4}$, the weight coefficient $aif = 2.75$, the local control rule is the linear control rule, the linear weight coefficient $C_{p} = 0.225$.

**Figure 23:**
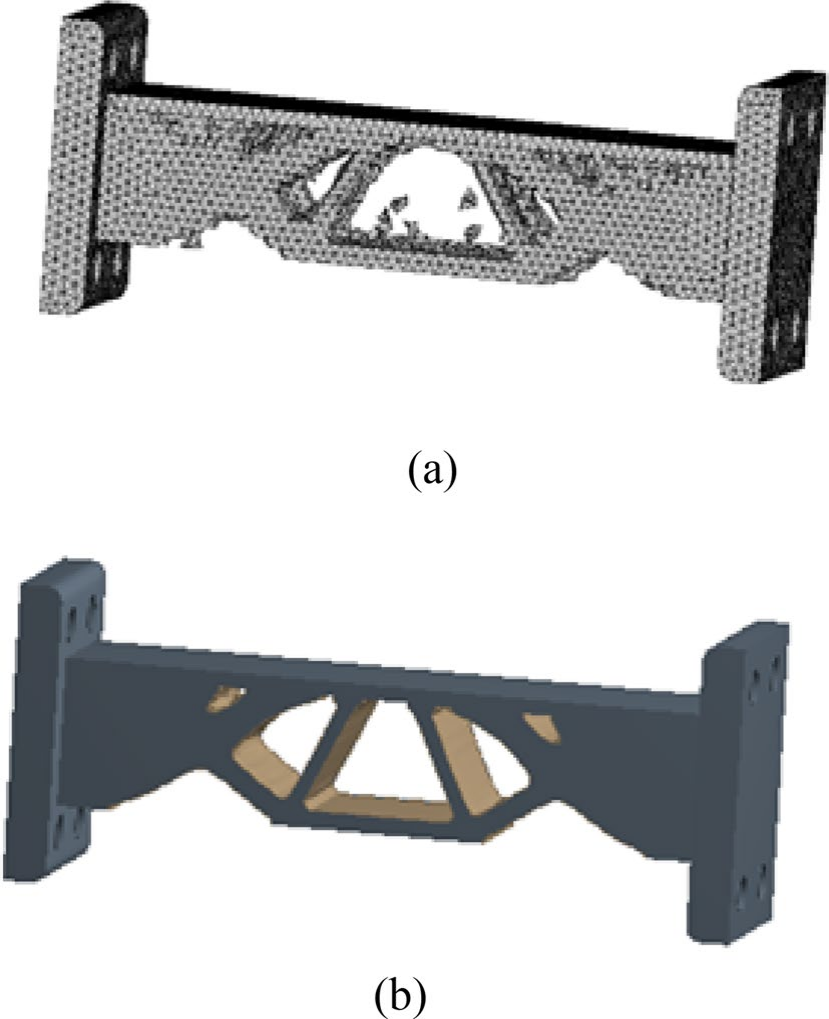
Comparison of optimization results with different optimization algorithms. (**a**) HCAM optimization result (**b**) FEM optimization result.

After HCAM calculation, the number of iterations to finally solve the result is 52, and the final topology optimization configuration obtained is shown in Fig. 23a, which is similar to the result obtained by ANSYS in Fig. 23b.

**Figure 24:**
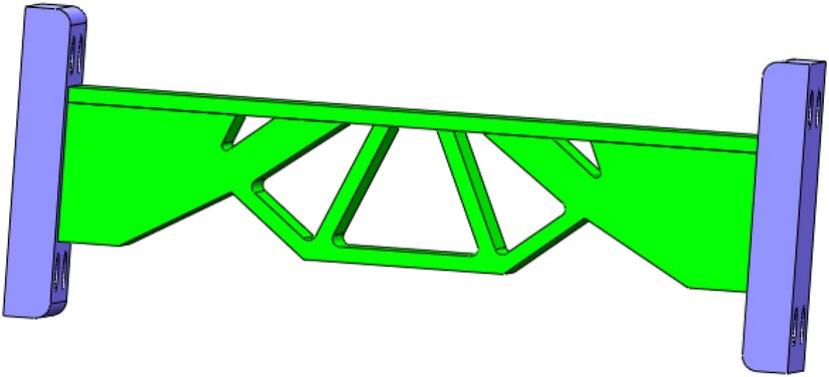
CAD software reconstructed model.

The obtained multi-material and multi-body topology optimization configuration is output to CAD software for geometric reconstructed of the model. The reconstructed model basically kept the original topology structure of the optimization result, as shown in Fig. 24.

**Figure 25:**
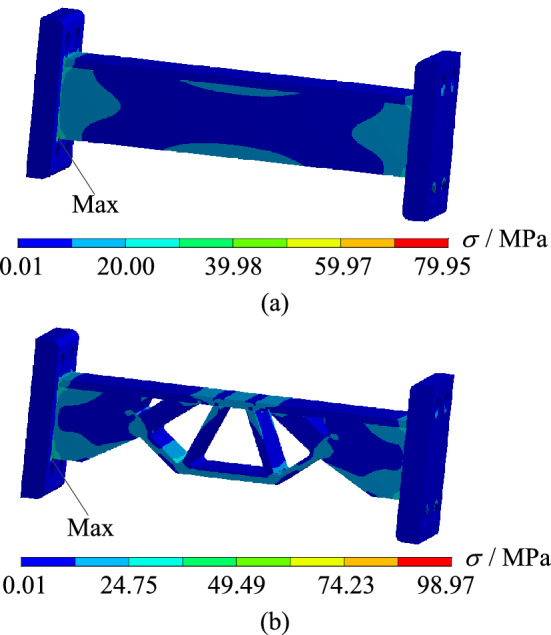
Comparison of stress contour figures before and after optimization. (**a**) Stress contour figure of the original model (**b**) Stress contour figure of the reconstructed model.

**Figure 26:**
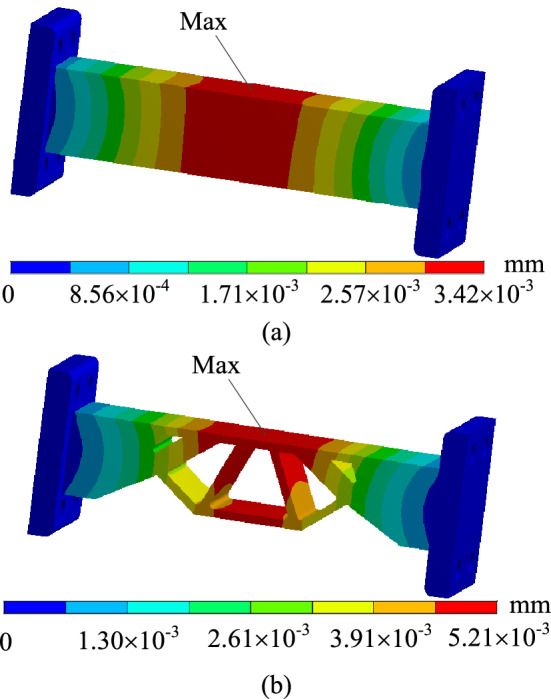
Comparison of displacement contour figures before and after optimization. (**a**) Displacement contour figure of the original model (**b**) Displacement contour figure of the reconstructed model.

In order to verify whether the geometrically reconstructed model can meet the requirements of working conditions, static simulation calculation is carried out under the same boundary conditions, and the stress and displacement comparison results are obtained as shown in Figs. 25 and 26. As can be seen from the stress contour figures in Fig. 25, the maximum stress in both the original model and the reconstructed model occurs below the contact position between connecting rod C and support A. The maximum stress of the original model is 79.95 MPa, while the maximum stress of the reconstructed model is 98.97 MPa. When 40% material is removed, the maximum stress increases by 23.80%, but the yield limit of connecting rod C material is 250 MPa, and the safety factor *n* is 2, which can still meet the strength requirements. At the same time, the maximum stress value of the non-design domain support A and B is 32.89 MPa, which is far lower than the material yield limit of the two supports 280 MPa, so the support-connecting rod-support body structure meets the strength requirements on the whole. Figure 26 is the displacement contour figure of the structure before and after optimization. It can be seen from the figure that the maximum displacement positions of before and after optimization are basically consistent, which all appear in the middle part of connecting rod C, which are $3.42 \times {10}^{ - 3} {\text{ mm}}$ and $5.21 \times {10}^{ - 3} {\text{ mm}}$, respectively.

## Concluding remarks

In this paper, a topology optimization design method for 3D multi-material and multi-body structures was proposed. The 3D tetrahedral cells with adaptive side length change are used to replace the traditional regular cube cells of HCAM, which can more flexibly adapt to complex geometric shapes. This avoids the problem that the cubic cells cannot uniformly cover the design area, so that more flexible solutions can be obtained when solving the topology configuration. At the same time, in order to solve the problem of contact relationship between objects in the multi-body structure, the contact problem processing method of FEM is applied to HCAM topology optimization, and the structure optimization configuration which is close to the existing commercial software module is obtained. Through the verification of typical numerical examples, the 3D multi-material and multi-body topology optimization method based on HCA can effectively remove the redundant materials of multi-material and multi-body structure, and the optimized structure configuration can satisfy the original condition requirement after geometrical reconstruction.
